# EVALUATING STOCHASTIC EPIGENETIC MUTATIONS: RELIABILITY, SUBTYPES, AND AN ALTERNATIVE DETECTION APPROACH

**DOI:** 10.1093/geroni/igad104.3309

**Published:** 2023-12-21

**Authors:** Yaroslav Markov, Albert Higgins-Chen

**Affiliations:** Yale Graduate School of Arts & Sciences, New Haven, Connecticut, United States; Yale School of Medicine, New Haven, Connecticut, United States

## Abstract

Stochastic Epigenetic Mutations (SEMs) have emerged as promising biomarkers for aging and age-related disorders. Conceptually, a SEM is defined as an outlier in DNA methylation (DNAme) value at a specific genomic site, compared to a given population or cohort. These mutations are seldom shared among subjects, emphasizing their potential to capture the heterogeneity of age-associated DNAme changes, and their cumulative count in individuals rises with age. We hypothesized that the original statistical method for SEM detection is not well suited for DNAme data and demonstrated its reliability constraints at both the individual and aggregate levels, using technical replicates from whole blood samples. In response to these challenges, we explored the factors influencing individual SEM reliability and identified best practices for SEM detection. We also developed an alternative machine learning-based detection method. Importantly, our classifier demonstrated improvements in the proportion of shared SEM and ICC scores when tested in an independent dataset with technical replicates, maintaining the associations of higher SEM loads with age, mortality, and cardiovascular disorders in the FHS study cohort. To support the wider application of our findings and address the need for SEM analysis tools, we have introduced a novel R package, SEMdetectR. Leveraging parallel programming, this package offers rapid SEM identification using both the original and new methods, accompanied by robust downstream analysis features.

